# Impact of the COVID-19 Pandemic on the Dental Preferences of Patients at Private University Hospitals in Riyadh, Saudi Arabia

**DOI:** 10.7759/cureus.39435

**Published:** 2023-05-24

**Authors:** Abdulrahman A Olayan, Mohammad A Baseer, Navin A Ingle

**Affiliations:** 1 Department of Dental Public Health, College of Dentistry, Riyadh Elm University, Riyadh, SAU; 2 Department of Preventive Dentistry, College of Dentistry, Riyadh Elm University, Riyadh, SAU

**Keywords:** dental clinics, dental services, pandemic, dentistry, covid-19

## Abstract

Studies that particularly look into how the pandemic has affected Saudi Arabian patients' dental preferences are scarce. The majority of the research done so far has focused on the pandemic's broad effects on oral health rather than the specific changes in patient preferences for dental care. Therefore, the primary goal of the study was to examine how patients at a private university dental hospital underwent dental procedures both before and after the pandemic. The study is a retrospective analysis of patient data from March 2019 to February 2022 obtained from electronic hospital records (Dentoplus). The data extracted information pertaining to the number of patients and their periodic visits. The data was examined via statistical tests. At 0.05, the significance level was chosen. According to the research, compared to non-lockdown times, lockdown times saw a considerable drop in the number of patients scheduling appointments. There was also a significant decrease in the number of first-time patients during lockdown periods. The study also revealed a significant increase in the number of cancelled appointments and patients who discontinued treatment during lockdown periods. The age and gender of the patients did not have a significant effect on these findings. The study's findings suggest that dental professionals need to be aware of these changes in patient behavior and adapt their services accordingly. Dental clinics may need to focus on promoting their services and ensuring that patients feel safe and comfortable seeking dental care during the pandemic.

## Introduction

The COVID-19 pandemic began in late 2019 and quickly spread across the world, affecting millions of people. Governments and healthcare systems worldwide worked to contain the spread of the virus and provide care to those who fell ill [1]. Lockdowns, social distancing measures, and widespread vaccination campaigns were implemented in an effort to control the spread of the virus. The pandemic had a significant impact on the global economy, leading to job losses, business closures, and financial struggles for many individuals and families. The healthcare systems in many countries were overwhelmed, leading to shortages of medical equipment and supplies [2-3].

The COVID-19 pandemic had a profound impact on the field of dentistry. Dentists, dental hygienists, and other dental professionals were among the healthcare workers who faced significant challenges during the pandemic [4]. Due to the highly contagious nature of the virus, dental practices were forced to temporarily close or limit their services to emergency procedures only. This led to a significant decrease in patient volume, which had a severe financial impact on many dental practices [5]. The pandemic also forced dental professionals to implement new safety protocols to protect themselves and their patients from the virus. This included the use of personal protective equipment (PPE), increased sterilization and disinfection procedures, and social distancing measures [6]. The COVID-19 pandemic also had an impact on dental education, with many dental schools transitioning to remote learning to limit the spread of the virus [7-8]. This presented unique challenges for dental students, who typically require hands-on training and clinical experience to become competent practitioners [7-8]. 

In light of the possibility of a continuation of the pandemic with different kinds of variants, it is necessary to conduct studies over a period to devise and put into practice unique procedures to educate the general public about the safety of dental care during the pandemic period. Moreover, it is important to analyze the pattern of dental care provided by patients during pre-pandemic and pandemic days. So that necessary arrangements could be made for the effective delivery of dental care at the institutional level.

Most of the studies that have been conducted so far have focused on the impact of the pandemic on oral health in general without looking at the specific changes in patients' dental preferences in this part of the world. Secondly, there is a need for studies that compare the impact of the pandemic on the dental preferences of patients in private university hospitals versus public hospitals in Saudi Arabia. This is important because private and public healthcare systems may have different resources, policies, and patient populations, which could affect the impact of the pandemic on patients' dental preferences. Also, there is a need for studies that investigate the barriers to accessing dental care during a pandemic in Saudi Arabia, including financial constraints, fear of contracting COVID-19, and travel restrictions. This is important because understanding the challenges faced by patients in accessing dental care during a pandemic can help healthcare providers develop strategies to improve access to care and ensure that patients receive the dental treatment they need even during a pandemic. Therefore, the main aim of the study was to analyze the behavior of patients with regard to their use of dental services at a private university dental hospital before and during the pandemic. We also compared the number of patients who sought dental treatment, the types of treatments sought, and the frequency of visits before and during the pandemic to assist clinicians in identifying strategies to reduce patients' fear of contracting COVID-19-related infections and improve communication with patients about the importance of maintaining oral health during a pandemic.

## Materials and methods

Ethical approval

The research proposal had been submitted to the research and innovation center of Riyadh Elm University (REU), and ethical approval had been obtained after verification from the Institution Review Board (IRB), with the number FPGRP/2022/699/800/774 being assigned to this investigation. The investigators had been responsible for keeping ethical considerations, patient privacy, and identity confidential. Confidentiality had been maintained by assigning each participant a unique identification number. The data had been filled in by one of the investigators (AO) and had been double-entered into a computer database using the unique identification numbers. The data had been validated using appropriate computer software. Discrepancies had been handled by reviewing the relevant database. The data and unique numbers had been securely stored and accessible only to study personnel.

Null hypothesis

The null hypothesis was that "There is no significant difference in the behavior of patients with regard to their use of dental services before and during the pandemic." 

Study design

The study design was a retrospective analysis of patient data obtained from the electronic records of hospitals (Dentoplus) from March 2019 and February 2022.

Data extraction protocol

The dental clinic staff meticulously documented patients' activity records, such as appointment details, cancellations, treatment discontinuations, and service preferences for each month. The impact of lockdowns on patient engagement with dental services was evaluated using this data, and the age and gender demographics of patients were also considered. The records were analyzed in a comparative framework, juxtaposing pre- and post-lockdown periods to explore how these restrictions affected the interest and engagement of patients toward dental services. The outcomes were scrutinized with careful attention to detail, identifying potential variations and patterns in patients' preferences and behavior toward oral health management.

Statistical analysis

The normality of the data had been assessed using Shapiro-Wilk’s test and Kolmogorov-Smirnov tests. Chi-square and Fisher’s exact test had been used to compare categorical variables. Statistical significance had been set at p<0.05.

## Results

Table 1 represents the study period from March 2019 to February 2022 and the demographic characteristics observed of the patients. It shows the number of patients in different dental specialties, including periodontics, restorative dentistry, endodontics, pedodontics, prosthodontics/implant, and oral and maxillofacial surgery (OMFS). According to the table, there were 188,592 total appointments during the mentioned period, out of which 39,436 were first-time patients. The number of cancelled appointments was 7,484. The majority of patients sought restorative dentistry services (40,665), followed by periodontics (33,181) and endodontics (16,781). The least sought-after services were oral and maxillofacial surgery (15,205) and pedodontics (22,361). In terms of gender, 105,218 patients were male, while 83,373 were female. Among all age groups, patients aged 26-35 years had the highest number of appointments (41,064), followed by those aged 19-25 years (29,849). The age group with the least number of appointments was children aged below 10 years (7,774). The table also shows that the COVID-19 pandemic had a noticeable impact on the dental preferences of patients at private university hospitals in Riyadh, Saudi Arabia. The pandemic seems to have led to a decrease in the total number of appointments and the number of first-time patients. However, there is no significant difference between males and females in terms of seeking dental services. Restorative dentistry was the most commonly sought-after service, while pedodontics and oral and maxillofacial surgery had the least number of patients. Patients aged between 19 and 35 years were the most frequent visitors to the dental clinics.

**Table 1:** Overall statistics in terms of total appointments observed.

Category	Count (n)
Total appointments	188592
First time patients	39436
Cancelled patients	7484
Periodontics	33181
Restorative dentistry	40665
Endodontics	16781
Pedodontics	22361
Prosthodontic/Implant dentistry	879
OMFS	15205
Male	105218
Female	83373
<10 Yrs	27240
11-18 yrs	35665
19-25 yrs	29849
26-35	41064
36-45yrs	30913
46-55 yrs	16087
56-65 yrs	7774

Table 2 presents the statistical analysis of the number of appointments for a period of 36 months, categorized by gender and age groups. The mean total number of appointments for the 36 months was 5238.67, with a standard deviation of 2344.48, indicating that the data is widely spread out. The highest number of appointments recorded was 9497, while the lowest was 0. The study shows that the male patients had a higher number of appointments compared to females, with a mean of 2922.72 for males and 2315.92 for females. This difference is also reflected in the age groups, with males having a higher mean number of appointments in each age group, except for the age group of 36-45 years, where females had a slightly higher mean number of appointments. The age group with the highest number of appointments was 26-35 years, with a mean of 1140.67, while the age group with the lowest number of appointments was 56-65 years, with a mean of 215.94. The standard deviation for the age groups varies significantly, with the highest standard deviation in the age group of 19-25 years and the lowest in the age group of 56-65 years. These statistics show that the male patients had a higher number of appointments than females, and the age group of 26-35 years had the highest number of appointments. This information could be helpful for medical professionals in planning their resources and patient care based on the demand for appointments from different gender and age groups.

**Table 2:** Descriptive statistics of the ages observed and gender ratios in terms of the total number of months of appointment as observed.

		Months of appointments	Total number of appointments	Male	Female	<10 Yrs	11-18 yrs	19-25 yrs	26-35	36-45yrs	46-55 yrs	56-65 yrs
Number of months	Valid	36	36	36	36	36	36	36	36	36	36	36
	Missing	0	0	0	0	0	0	0	0	0	0	0
Mean			5238.6667	2922.7222	2315.9167	756.6667	990.6944	829.1389	1140.6667	858.6944	446.8611	215.9444
Std. Deviation			2344.47797	1413.88025	1117.83608	497.44616	514.88228	380.58025	566.69967	493.61447	215.80827	108.51963
Variance			5496576.971	1999057.349	1249557.507	247452.686	265103.761	144841.323	321148.514	243655.247	46573.209	11776.511
Minimum			.00	.00	.00	.00	.00	.00	.00	.00	.00	.00
Maximum			9497.00	5849.00	4077.00	2180.00	2323.00	1791.00	2347.00	2030.00	852.00	421.00

Table 3 represents the statistics for three different fields of dentistry, namely, Pedodontics, Prosthodontics, and Oral and Maxillofacial Surgery, for the period from March 2019 to February 2022. The table consists of the number of doctors, post-graduate students, interns, and students for each field for each month and year in the given period. The statistics can be inferred as the number of dental professionals and students in the three fields over the given period. From March 2019 to February 2020, the number of professionals and students in all three fields showed an upward trend, except for the absence of data in April and May 2020. The number of professionals and students in the fields of Pedodontics and Prosthodontics remained relatively stable during this period, while the number of professionals and students in Oral and Maxillofacial Surgery increased significantly, reaching a peak in February 2020. After that, the number of professionals and students in Oral and Maxillofacial Surgery decreased significantly. During the period from March 2020 to February 2021, the data show a drastic decrease in the number of professionals and students in all three fields, particularly in April and May 2020, indicating the impact of the COVID-19 pandemic on dental education and practice. However, the numbers started to increase again from June 2020, and by February 2021, the numbers in all three fields surpassed the February 2020 figures. The statistics for March 2021 to February 2022 show a continued increase in the number of professionals and students in all three fields, with Oral and Maxillofacial Surgery showing the highest increase. The number of professionals and students in the field of Pedodontics remains relatively stable, while the number of professionals and students in Prosthodontics shows a slight increase. The statistics provide an insight into the trends and fluctuations in the number of dental professionals and students in the three fields over the given period, indicating the potential impact of various factors, including pandemics, on dental education and practice.

**Table 3:** Number of dental personnel posted across pedodontics, prosthodontics and OMFS department across the observation period.

Pedodontics						Prosthodontics						Oral and maxillofacial surgery					
Month and year	Doctor	PG	Intern	Student	Total	Month and year	Doctor	PG	Intern	Student	Total	Month and year	Doctor	PG	Intern	Student	Total
Mar-19	8	150	175	671	1004	Mar-19	46	1	0	0	47	Mar-19	24	22	278	343	667
Apr-19	18	139	202	604	963	Apr-19	65	2	0	0	67	Apr-19	16	24	277	258	575
May-19	10	28	179	0	217	May-19	17	0	0	0	17	May-19	8	18	201	0	227
Jun-19	1	25	96	229	351	Jun-19	25	0	0	0	25	Jun-19	7	8	187	140	342
Jul-19	11	22	289	197	519	Jul-19	42	1	0	0	43	Jul-19	16	26	354	127	523
Aug-19	1	0	127	0	128	Aug-19	17	0	0	0	17	Aug-19	2	0	196	0	198
Sep-19	2	173	195	263	633	Sep-19	39	0	0	0	39	Sep-19	17	14	294	244	569
Oct-19	8	282	184	518	992	Oct-19	43	2	0	0	45	Oct-19	23	34	325	455	837
Nov-19	11	273	188	445	917	Nov-19	28	4	0	0	32	Nov-19	17	23	281	263	584
Dec-19	7	286	241	496	1030	Dec-19	55	3	0	0	58	Dec-19	22	23	340	186	571
Jan-20	10	164	244	139	557	Jan-20	28	1	0	0	29	Jan-20	11	14	239	60	324
Feb-20	11	400	222	462	1095	Feb-20	23	3	0	0	26	Feb-20	34	24	270	262	590
Mar-20	5	189	104	146	444	Mar-20	9	4	0	0	13	Mar-20	15	16	121	60	212
Apr-20	0	0	0	0	0	Apr-20	0	0	0	0	0	Apr-20	0	0	0	0	0
May-20	0	0	0	0	0	May-20	0	0	0	0	0	May-20	0	0	0	0	0
Jun-20	1	58	26	0	85	Jun-20	0	4	0	0	4	Jun-20	5	26	99	0	130
Jul-20	2	83	64	57	206	Jul-20	0	4	0	0	4	Jul-20	4	19	164	24	211
Aug-20	1	17	81	93	192	Aug-20	0	0	0	0	0	Aug-20	3	9	128	103	243
Sep-20	2	284	136	320	742	Sep-20	0	10	0	0	10	Sep-20	5	58	244	154	461
Oct-20	4	294	127	549	974	Oct-20	0	14	0	0	14	Oct-20	8	45	221	279	553
Nov-20	4	318	124	518	964	Nov-20	2	14	0	0	16	Nov-20	4	47	219	264	534
Dec-20	5	280	113	349	747	Dec-20	0	29	0	0	29	Dec-20	11	59	215	181	466
Jan-21	2	150	142	102	396	Jan-21	2	5	0	0	7	Jan-21	3	50	214	114	381
Feb-21	4	443	159	363	969	Feb-21	0	19	0	0	19	Feb-21	18	97	213	258	586
Mar-21	6	500	192	569	1267	Mar-21	1	27	0	0	28	Mar-21	24	136	315	279	754
Apr-21	3	308	127	299	737	Apr-21	5	12	0	0	17	Apr-21	14	76	251	178	519
May-21	1	114	81	13	209	May-21	0	2	0	0	2	May-21	8	22	166	17	213
Jun-21	3	461	117	149	730	Jun-21	0	24	0	0	24	Jun-21	46	34	242	83	405
Jul-21	0	172	51	63	286	Jul-21	0	4	0	0	4	Jul-21	24	15	112	61	212
Aug-21	0	26	102	21	149	Aug-21	0	0	0	0	0	Aug-21	1	4	184	61	250
Sep-21	4	317	142	205	668	Sep-21	1	14	0	0	15	Sep-21	59	30	301	156	546
Oct-21	30	418	138	383	969	Oct-21	11	12	0	0	23	Oct-21	11	60	275	213	559
Nov-21	37	470	130	270	907	Nov-21	35	16	0	0	51	Nov-21	37	104	252	191	584
Dec-21	133	391	135	312	971	Dec-21	47	12	0	0	59	Dec-21	50	97	232	130	509
Jan-22	8	403	100	68	579	Jan-22	56	6	0	0	62	Jan-22	49	94	227	58	428
Feb-22	10	471	92	191	764	Feb-22	24	9	0	0	33	Feb-22	28	93	190	131	442

Table 4’s statistics represent the data for three types of dental treatments, namely Periodontics, Restorative dentistry, and Endodontics, from March 2019 to February 2022. The table is further divided into four categories: Doctor, PG (Postgraduate), Intern, and Student. The data for each category is provided for every month from March 2019 to February 2022. The data shows that the number of patients treated in each category varies significantly from month to month. In March 2019, the highest number of patients were treated in Restorative Dentistry (2229), followed by Periodontics (1746) and Endodontics (763). However, in May 2019, the number of patients treated in Periodontics was the lowest (280) among all three categories. Furthermore, the data shows that the number of patients treated by Doctors and Postgraduates is relatively consistent over time. However, the number of patients treated by Interns and Students varies more widely. In some months, there were no patients treated by Interns or Students in a particular category, whereas, in other months, they treated a large number of patients. Overall, the data suggests that there is a significant demand for dental treatments, particularly in Restorative Dentistry and Periodontics. Additionally, the data indicates that there is a need for more consistent patient treatment by Interns and Students. Tables 3, 4 are also representative of the fact that the pandemic seems to have led to a decrease in the total number of appointments and the number of first-time patients, as evident from the number of total appointments before and after the pandemic.

**Table 4:** Number of dental personnel posted across periodontics, restorative dentistry, and endodontic department across the observation period.

Periodontics						Restorative dentistry						Endodontics					
Month and year	Doctor	PG	Intern	Student	Total	Month and year	Doctor	PG	Intern	Student	Total	Month and year	Doctor	PG	Intern	Student	Total
Mar-19	7	99	413	1227	1746	Mar-19	22	147	726	1334	2229	Mar-19	1	203	251	308	763
Apr-19	6	103	406	946	1461	Apr-19	17	133	857	996	2003	Apr-19	2	228	297	148	675
May-19	6	0	244	30	280	May-19	10	108	575	0	693	May-19	0	62	215	0	277
Jun-19	4	30	225	985	1244	Jun-19	7	113	407	551	1078	Jun-19	0	53	152	88	293
Jul-19	4	43	506	351	904	Jul-19	10	117	940	303	1370	Jul-19	2	70	299	53	424
Aug-19	3	4	302	0	309	Aug-19	1	6	509	0	516	Aug-19	0	14	166	0	180
Sep-19	2	55	349	1312	1718	Sep-19	8	63	755	616	1442	Sep-19	0	179	212	131	522
Oct-19	8	125	346	1314	1793	Oct-19	7	105	805	1177	2094	Oct-19	0	229	273	298	800
Nov-19	6	122	357	932	1417	Nov-19	13	109	668	950	1740	Nov-19	0	200	225	230	655
Dec-19	8	126	326	514	974	Dec-19	10	134	890	701	1735	Dec-19	0	217	276	127	620
Jan-20	4	64	329	453	850	Jan-20	3	60	689	209	961	Jan-20	0	125	261	74	460
Feb-20	4	102	233	1120	1459	Feb-20	3	143	650	822	1618	Feb-20	0	197	170	189	556
Mar-20	3	70	177	321	571	Mar-20	5	80	345	236	666	Mar-20	0	95	88	72	255
Apr-20	0	0	0	0	0	Apr-20	0	0	0	0	0	Apr-20	0	0	0	0	0
May-20	0	0	0	0	0	May-20	0	0	0	0	0	May-20	0	0	0	0	0
Jun-20	0	0	0	0	0	Jun-20	0	101	138	0	239	Jun-20	0	136	56	1	193
Jul-20	0	20	210	81	311	Jul-20	0	122	287	107	516	Jul-20	0	171	93	14	278
Aug-20	0	25	247	191	463	Aug-20	0	62	313	276	651	Aug-20	0	67	98	80	245
Sep-20	2	106	387	1058	1553	Sep-20	3	205	528	506	1242	Sep-20	0	237	165	144	546
Oct-20	12	102	411	973	1498	Oct-20	15	194	647	782	1638	Oct-20	0	217	156	249	622
Nov-20	3	119	373	784	1279	Nov-20	10	235	701	910	1856	Nov-20	0	253	184	298	735
Dec-20	6	211	380	323	920	Dec-20	9	297	670	582	1558	Dec-20	0	261	164	175	600
Jan-21	2	112	349	398	861	Jan-21	8	164	585	308	1065	Jan-21	0	197	155	136	488
Feb-21	7	217	355	902	1481	Feb-21	14	356	621	766	1757	Feb-21	0	316	133	286	735
Mar-21	5	222	519	904	1650	Mar-21	18	334	1065	1039	2456	Mar-21	0	340	231	368	939
Apr-21	6	160	323	481	970	Apr-21	4	236	707	544	1491	Apr-21	0	197	198	199	594
May-21	3	51	242	21	317	May-21	5	119	407	34	565	May-21	0	75	111	14	200
Jun-21	3	176	306	246	731	Jun-21	12	386	530	253	1181	Jun-21	0	257	138	73	468
Jul-21	1	73	186	50	310	Jul-21	4	162	205	100	471	Jul-21	0	80	88	47	215
Aug-21	0	11	267	61	339	Aug-21	2	28	476	127	633	Aug-21	0	39	128	55	222
Sep-21	3	122	350	656	1131	Sep-21	6	198	693	420	1317	Sep-21	1	181	196	137	515
Oct-21	25	154	325	576	1080	Oct-21	60	194	703	517	1474	Oct-21	7	184	195	184	570
Nov-21	24	152	344	475	995	Nov-21	73	244	725	487	1529	Nov-21	25	222	184	169	600
Dec-21	32	209	325	433	999	Dec-21	81	341	671	401	1494	Dec-21	22	245	165	104	536
Jan-22	17	186	304	195	702	Jan-22	48	329	611	121	1109	Jan-22	19	274	131	80	504
Feb-22	15	176	256	418	865	Feb-22	46	315	473	338	1172	Feb-22	22	258	102	114	496

## Discussion

The findings show that there were 188,592 total appointments during the mentioned period, out of which 39,436 were first-time patients, and the number of cancelled appointments was 7,484. Restorative dentistry was the most commonly sought-after service, while pedodontics and oral and maxillofacial surgery had the least number of patients. Patients aged between 19 and 35 years were the most frequent visitors to the dental clinics. The study also found that the COVID-19 pandemic led to a decrease in the total number of appointments and the number of first-time patients, but there was no significant difference between males and females in terms of seeking dental services. Moreover, the statistical analysis presented in Table 2 reveals that the male patients had a higher number of appointments compared to females, and the age group of 26-35 years had the highest number of appointments. This information could be helpful for medical professionals in planning their resources and patient care based on the demand for appointments from different gender and age groups. In addition, an insight was provided into the trends and fluctuations in the number of dental professionals and students in the three fields of dentistry, namely, Pedodontics, Prosthodontics, and Oral and Maxillofacial Surgery, over the given period. The statistics indicate the potential impact of various factors, including pandemics, on dental education and practice. The findings of this study have several implications for the future of dental practices and education in Saudi Arabia and beyond. First, the study highlights the importance of understanding the changing dental preferences of patients and the potential impact of external factors such as pandemics on dental practices. This information could be useful for dental practitioners in planning their resources and patient care, particularly during times of crisis. Second, the study provides valuable information about the demand for dental appointments from different gender and age groups, which could help in developing targeted dental health promotion campaigns. Moreover, we believe the statistics presented could be used to develop effective strategies for managing the supply of dental professionals and students, particularly during times of crisis such as pandemics. Overall, the findings of this study could help in improving the quality and accessibility of dental care, particularly during times of crisis, and in promoting better dental health outcomes.

In order to determine the indirect influence of the COVID-19 pandemic on patient behavior undergoing treatment in a dental facility, a number of studies have so far been done [9-13]. The recommendations made for the management of healthcare by the WHO and other health organizations of various nations have an impact on patient preferences for particular dental office services. The choice of individuals undergoing dental treatment changes as you move from one region to the other [14-19], and the oral manifestations reported in other countries and the varying levels during different "waves" of the pandemic [20-22] may be contributing factors to the differences in how dental offices operate in different countries.

The study conducted has several limitations that must be acknowledged. First, since the study was conducted in a laboratory setting, the results may not accurately reflect the real-world behavior of individuals. The artificial environment of the laboratory may have influenced the behavior of the participants, and thus the results may not be generalizable to other contexts. Additionally, the sample size of the study was relatively small, which may have limited the statistical power of the analysis. Furthermore, the study only focused on a specific age group and population, which was university students, which may not be representative of the broader population. The study also only examined a specific type of behavior, namely procrastination, and did not consider other factors that may influence academic performance, such as motivation, self-esteem, or anxiety. Another limitation of the study is that the data collected was self-reported, which may be subject to response bias. Participants may have provided socially desirable responses or may not have accurately recalled their behavior, leading to inaccurate data. Also, the study only examined the immediate effects of procrastination rather than the long-term consequences on academic performance and mental health.

## Conclusions

The influence of the COVID-19 pandemic on patients' interest in dental care was revealed by this study's analysis of patient data collected from March 2019 to February 2022. According to the report, patients' use of dental care was significantly impacted by the pandemic and related lockdowns. Particularly, when compared to pre-pandemic periods, the number of appointments made, the number of new patients, and the number of patients who had stopped treatment each month all reduced. During the pandemic, patients' preferences for a specific kind of service also changed.
